# Comparison of Conventional Molecular and Whole-Genome Sequencing Methods for Differentiating *Salmonella enterica* Serovar Schwarzengrund Isolates Obtained from Food and Animal Sources

**DOI:** 10.3390/microorganisms9102046

**Published:** 2021-09-28

**Authors:** I-Chen Li, Rayean Wu, Chung-Wen Hu, Keh-Ming Wu, Zeng-Weng Chen, Chung-Hsi Chou

**Affiliations:** 1 Zoonoses Research Center, School of Veterinary Medicine, National Taiwan University, No. 1, Sec. 4, Roosevelt Rd., Taipei City 106, Taiwan; d06629004@ntu.edu.tw (I.-C.L.); rayeanwu@gmail.com (R.W.); charlotte.hu@gmail.com (C.-W.H.); 2 Welgene Biotech Co., Ltd., 12F, No. 3, Park St., Taipei City 115, Taiwan; kmwu@welgene.com.tw; 3 Animal Technology Research Center, Agricultural Technology Research Institute, No. 52, Kedong 2nd Rd., Zhunan Township, Miaoli County 350, Taiwan

**Keywords:** *Salmonella*, subtyping, PFGE, MLST, CRISPR, WGS

## Abstract

Over the last decade, *Salmonella* *enterica* serovar Schwarzengrund has become more prevalent in Asia, Europe, and the US with the simultaneous emergence of multidrug-resistant isolates. As these pathogens are responsible for many sporadic illnesses and chronic complications, as well as outbreaks over many countries, improved surveillance is urgently needed. For 20 years, pulsed-field gel electrophoresis (PFGE) has been the gold standard for determining bacterial relatedness by targeting genome-wide restriction enzyme polymorphisms. Despite its utility, recent studies have reported that PFGE results correlate poorly with that of closely related outbreak strains and clonally dominant endemic strains. Due to these concerns, alternative amplification-based molecular methods for bacterial strain typing have been developed, including clustered regular interspaced short palindromic repeats (CRISPR) and multilocus sequence typing (MLST). Furthermore, as the cost of sequencing continues to decrease, whole genome sequencing (WGS) is poised to replace other molecular strain typing methods. In this study, we assessed the discriminatory power of PFGE, CRISPR, MLST, and WGS methods to differentiate between 23 epidemiologically unrelated *S. enterica* serovar Schwarzengrund isolates collected over an 18-year period from distinct locations in Taiwan. The discriminatory index (DI) of each method for different isolates was calculated, resulting in values between 0 (not discriminatory) and 1 (highly discriminatory). Our results showed that WGS has the greatest resolution (DI = 0.982) compared to PFGE (DI = 0.938), CRISPR (DI = 0.906), and MLST (DI = 0.463) methods. In conclusion, the WGS typing approach was shown to be the most sensitive for *S. enterica* serovar Schwarzengrund fingerprinting.

## 1. Introduction

Salmonellosis is a public health concern in both industrialized and developing countries. Nontyphoidal *Salmonella* serovars cause high morbidity and mortality, as well as considerable global economic loss each year [1]. In humans, salmonellosis is characterized by self-limiting gastroenteritis, and usually results from consuming contaminated animal products, such as poultry, meat, eggs, or dairy [2]. As *Salmonella* can infect human hosts by different sources, accurately identifying and discriminating between isolates is important for global public health authorities to detect outbreaks and track the originating sources [3]. For over 50 years, surveillance data from the Centers for Disease Control and Prevention (CDC) for *Salmonella* serotype designation have been collected through laboratory-based surveillance systems [4]. However, conventional serotyping based on antisera agglutination has several limitations as it is not discriminative enough to infer phylogenetic relationships [5]. Since there has been a tremendous improvement in bacterial subtyping techniques over the last decade [6], the need for a better *Salmonella* surveillance system is required.

Alternative methods such as pulsed-field gel electrophoresis (PFGE), multilocus sequence typing (MLST), clustered regularly interspaced short palindromic repeats (CRISPR), and whole genome sequencing (WGS), have been proposed to replace conventional *Salmonella* serotyping methods [6]. PFGE involves the macrorestriction of genomic DNA by specific enzymes, the separation of fragments by a pulsed electric field, and an analysis of the resulting DNA fingerprint [7]. Conversely, MLST directly measures the genetic variations in seven housekeeping genes and defines strains based on their unique allelic profiles [8]. CRISPR compares spacers of CRISPR loci and cluster strains based on their spacer content similarity [9]. Lastly, WGS differentiates virtually all strains by detecting variation across the complete bacterial genome [10]. Although each method has advantages and disadvantages regarding speed, cost, strength, and sensitivity [11], these techniques can identify geographically dispersed outbreaks at an earlier stage [12].

Among genotype approaches, PFGE is the most commonly used molecular typing method and remains a gold standard method for the identification of foodborne pathogens tracked by PulseNet, a global laboratory network comprising 86 countries [13]. While PFGE is robust and reliable, in some cases it does not generate sufficient discrimination, particularly for closely related isolates [14]. To enhance resolution, MLST was introduced and has been useful in distinguishing isolates sharing apparently identical PFGE profiles [15]. Additionally, CRISPR was proposed and found to be more discriminatory than PFGE analysis for the same group of prevalent MDR DT104 isolates (Discriminatory index (DI) = 0.64 vs. DI = 0.38) [9]. Moreover, as WGS could provide the entire genetic blueprint of a pathogen, it has become an increasingly popular method for use in public health laboratories [16,17]. Depending on the research question, WGS not only discriminates between isolates at the Single Nucleotide Polymorphism (SNP) level, a transmission pathway of pathogens, but also offers the possibility to detect the presence of virulence and antibiotic-resistance genes [18]. However, although WGS has the capacity to replace other existing conventional typing methods, validation studies are needed to ensure the robustness and technical performance of this approach. Therefore, after this study is performed, the results should verify the performance of WGS as superior to other existing molecular subtyping methods for *Salmonella* surveillance, and further strengthen the implementation of WGS to track foodborne pathogens on a global scale.

Among many *Salmonella enterica* serovars, serovar Schwarzengrund is responsible for human and poultry infections in Asia and some Western nations [19,20]. The reported sources of these outbreaks have been ground turkey, chicken meat, dry dog food, and cat food [21,22]. Not only have *S. enterica* serovar Schwarzengrund strains spread from food to humans, these strains also display multi-antibiotic resistance and produce extended-spectrum *β*-lactamases, including carbapenemase [19,20]. However, although *S. enterica* serovar Schwarzengrund strains have been isolated consistently, few genotyping studies have been carried out. A suitable *Salmonella* typing technique could increase the understanding of disease pathogenesis, transmission, and prevention. Therefore, the aim of the present study was to compare four different molecular *Salmonella* subtyping methods to discriminate *S. enterica* serovar Schwarzengrund.

## 2. Materials and Methods

### 2.1. Bacterial Isolates

A total of 23 epidemiologically unrelated, and one related, *S. enterica* serovar Schwarzengrund strains collected between 2000 and 2018 were analyzed in this study (Table 1). Serotyping was performed using the White–Kauffmann–Le Minor scheme as previously described [23]. These isolates were obtained from multiple sources, including duck (*n* = 2), pig (*n* = 7), dog (*n* = 1), broiler (*n* = 8), pet food (*n* = 1), crested goshawk (*n* = 1), moorhen (*n* = 1), and turkey (*n* = 3). Among these strains, SS07 came from the same source as SS08 and was included to serve as an internal control for comparison. All bacteria were grown on trypticase soy agar or in trypticase soy broth, and incubated aerobically at 37 °C for 12 h before molecular characterization.

### 2.2. PFGE

PFGE was performed according to PulseNet protocol developed by the CDC [24]. *S. enterica* serovar Braenderup (ATCC BAA 664) was used as the control strain. Briefly, agarose plugs containing genomic DNA treated with Proteinase K Buffer were incubated with 20 units of *Xba*I enzyme at 37 °C for 2 h (New England BioLabs, Ipswich, MA, USA). DNA separation was performed with 1% SeaKem Gold agarose gels in 0.5 M Tris borate–EDTA buffer at 14 °C for 18 h with pulse times between 2.16 and 63.8 s using a CHEF DR III apparatus (Bio-Rad, Hercules, CA, USA). Gels were stained with ethidium bromide, visualized under UV light, and photographed. DNA fingerprints were analyzed using Bionumerics 7.1 software (Applied Maths, Austin, TX, USA). A phylogenetic tree was constructed using the Unweighted Pair Group Method, with Arithmetic Mean method with Dice coefficient at an optimization setting of 1% and a position tolerance setting of 1.5%, as recommended previously [25].

### 2.3. MLST

Bacterial DNA was isolated with a DNeasy^®^ Blood and Tissue kit (QIAGEN, Hilden, Germany) according to the manufacturer’s instructions, and quantified using a Biodrop Duo (Biochrom, Cambridge, United Kingdom). MLST was performed on seven gene fragments (*aroC*, *dnaN*, *hemD*, *hisD*, *purE*, *sucA*, and *thrA*; Table S1), as previously described [26]. In brief, 0.25 μM DNA template and 1 μM of each primer were added to 25 μL Taq DNA polymerase 2X Master Mix Red (Ampliqon, Bie and Berntsen, Herlev, Denmark) before amplification by a T-100 thermal cycler (Bio-Rad Laboratories, Hercules, CA, USA). PCR cycling parameters included denaturation at 94 °C for 10 min, followed by 25 cycles of 94 °C for 50 s, 57 °C for 50 s, 72 °C for 50 s, and elongation at 72 °C for 5 min. All PCR products were sequenced (Genomics Ltd., Taipei, Taiwan), purified, trimmed as indicated, and assigned to sequence types (ST) according to the MLST website (http://enterobase.warwick.ac.uk/species/senterica/allele_st_search, accessed on 15 June 2021).

### 2.4. CRISPR

Amplification of the CRISPR1 and CRISPR2 loci (Table S1) from *S. enterica* serovar Schwarzengrund was carried out according to the previous study [27]. In essence, PCR was performed in a reaction volume of 25 µL containing Taq DNA polymerase 2X Master Mix Red, 1 μM of each primer, and 0.25 μM DNA template. The cycling conditions were performed with initial denaturation of 95 °C for 10 min, followed by 45 cycles of 95 °C for 1 min, 55 °C for 90 s, 72 °C for 90 s, and final extension at 72 °C for 10 min. All PCR products were sequenced (PURIGO Biotechnology Ltd., Taipei, Taiwan) before CRISPR1 and CRISPR2 arrays were analyzed using CRISPR-finder (http://crispr.u-psud.fr/Server/, accessed on 15 June 2021). CRISPR types (CTs) were assigned based on the allelic profile (the combination of CRISPR 1 and CRISPR 2), as previously described [28].

### 2.5. WGS

Genomic DNA was extracted using DNeasy blood and tissue kit (Qiagen, CA, USA) according to the manufacturer’s instructions. For library construction, 3–5 μg total DNA was sonicated by a Misonix 3000 sonicator and checked by a DNA 1000 chip bioanalyzer (Agilent Technologies, Santa Clara, CA, USA) to generate DNA fragments between 180–200 base pairs (bp). One-point-five micrograms of sonicated DNA was end-repaired, A-tailed, and adaptor-ligated using the TruSeq DNA preparation kit (Illumina, San Diego, CA, USA) following the manufacturer’s guidelines. The prepared libraries were sequenced on the NextSeq500 platform (Illumina, Inc., San Diego, CA, USA) with 150PE protocol. The average sequencing depth of the libraries was 944.4 MegaBase (190× coverage). Illumina WGS data used in this study can be found under the NCBI BioProject accession PRJNA635494. The raw reads were trimmed and filtered using Trimmomatic software (version 0.36) developed by Bolger et al. [29]. Reads with average quality value ≥20 and read length ≥30 using default parameters were used for subsequent analysis.

The trimmed reads of each sample were de novo assembled into contigs using the SPAdes genome assembler (version 3.14.1) developed by Prjibelski et al. [30]. The assembled contigs of each sample were ordered, orientated and joined into single scaffold using MeDuSa developed by Bosi et al. [31], based on the reference genome sequence (*S. enterica* subsp. enterica serovar Schwarzengrund strain CVM19633 of the EnsemblBacteria database (http://bacteria.ensembl.org/index.html, accessed on 15 June 2021)). Pairwise comparisons of all assemblies against the reference with a default minimum mapping quality of 20 were performed using MUMmer3 software according to a previous study [32]. In brief, each pair of genome sequences were detected twice (reference vs. query, and the reciprocal order) and the list of maximal unique matches (MUMs) was generated using MUMmer3 software version 3.23 developed by Kurtz et al. with the following parameters: -mum, -b, -c, and -l 19 [33]. MUMmer3 results were parsed for non-overlapping MUMs, and then an average MUMi value was calculated for each pair of genomes using the following formula: MUMi = 1- Lmum/Lav, where Lmum is the sum of the length of all non-overlapping MUMs and Lav is the average length of the two genomes to be compared. These MUMi values were then outputted as a distance matrix file for constructing a phylogenetic tree using a neighbor-joining method and visualized using Molecular Evolutionary Genetics Analysis program software (version 7) developed by Kumar et al. [34].

### 2.6. Data Analysis

DI was used to assess the ability of a typing method to distinguish between strains. The discriminatory ability of each molecular typing system was evaluated by calculating the Simpson’s index of diversity as follows:

(1) $$\mathrm{DI}=1-\frac{1}{N\left(N-1\right)}\sum_{j=1}^{s}x_{j}(x_{j}-1)\;$$

where N is the number of unrelated strains tested, s is the number of different types, and x_j_ is the number of strains belonging to the jth type. The 95% confidence interval was calculated using the formulae described previously [35]. An ideal DI value would be at least 0.95 [36].

## 3. Results

**Figure 1:**
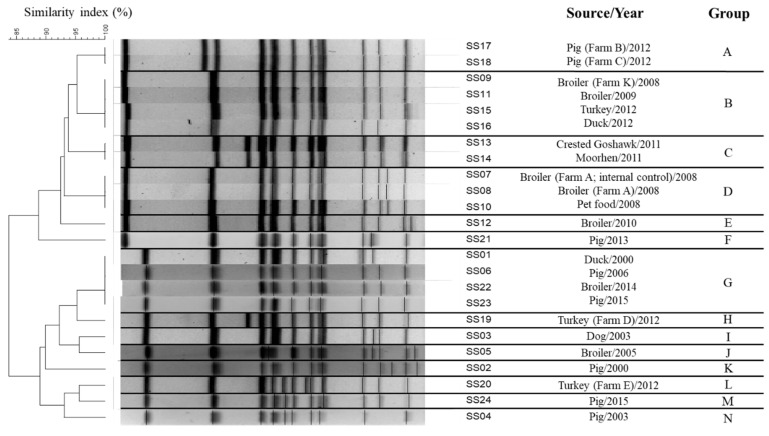
PFGE profiles of 24 *S. enterica* serovar S. Schwarzengrund isolates (14 profiles; DI = 0.938).

**Figure 2:**
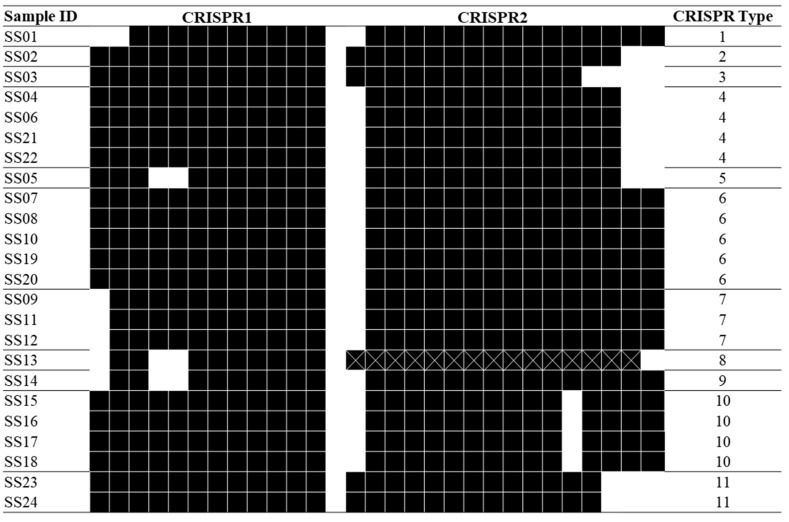
CRISPR spacer content of 24 *S. enterica* serovar Schwarzengrund isolates (11 types; DI = 0.906). Each unique spacer composition defines an allele, and a combination of CRISPR1 and CRISPR2 alleles was manually assigned an arbitrary CRISPR Type. Identical spacers shared between isolates under the same columns are shown as solid rectangles. Empty areas indicate that the corresponding spacer is not present in other similar patterns.

**Figure 3:**
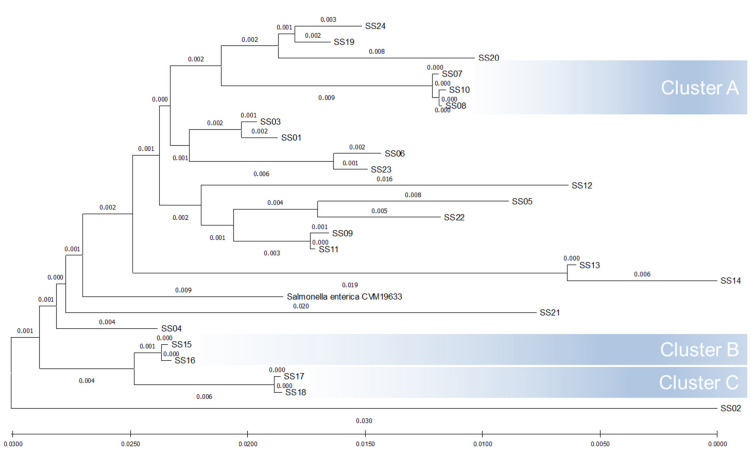
Comparative analysis of 24 *S. enterica* serovar Schwarzengrund isolates built using MUMi and neighbor-joining method (20 genotypes; DI = 0.982). The MUMi distance varies from 0, for very similar genomes, and 1, for very distant genomes.

By using the restriction enzyme *Xba*I, PFGE profiling generated from the 24 *S. enterica* serovar Schwarzengrund isolates identified 14 unique patterns (A through N) (Figure 1), and yielded a DI value of 0.938 (Table 2). Among the PFGE patterns, B and G were the most prevalent profiles, each consisting of four isolates, followed by pattern D, which included three isolates, and finally patterns A and C, which each had two isolates. The remaining nine strains isolated from different sources were clearly discriminated and sorted into different clades. Among the 24 isolates examined by MLST, 16 isolates were assigned to ST96, while eight isolates were clustered together into ST322, resulting in a DI value of 0.463 (Table 2). The thrA gene was found to be polymorphic among isolates (16 strains have allele type 3 and 8 strains have allele type 114). All isolates shared identical alleles at six of the MLST loci (aroC allele type 43, dnaN allele type 47, hemD allele type 49, hisD allele type 49, purE allele type 41, and sucA allele type 15). The observed CRISPR1 and CRISPR2 spacer contents of the 24 *S. enterica* serovar Schwarzengrund isolates are depicted in Figure 2. A maximum of 12 spacers were identified in the CRISPR1 locus, while a maximum of 15 spacers were identified in the CRISPR2 locus. All identified spacers in both CRISPR loci were 32 bp in length, and many spacers were shared among strains (Table S2). When combined, the CRISPR1 and CRISPR2 alleles constituted 11 different CTs among the 24 strains tested, which produced a DI value of 0.906. In the phylogenetic tree, generated based on DNA maximal unique matches (MUM), all 24 isolates formed a monophyletic group (Figure 3).

Pairwise whole genome sequences between the strains, calculated as the MUM index (MUMi), ranged from very similar genomes (0.000) to slightly divergent (0.030). A clear separation of different strains was observed, except for SS07, SS08, SS10, SS15, SS16, SS17, and SS18 strains, which were clustered close together into three different genetic groups. Twenty genotypes were then identified with a DI value of 0.982. The discriminatory index along with the corresponding confidence intervals for different subtyping methods among 24 *S. enterica* serovar Schwarzengrund isolates are shown in Table 2. WGS was the most discriminatory, differentiating the 24 *S. enterica* serovar Schwarzengrund strains into 20 types, followed by PFGE, CRISPR, and finally MLST methods.

## 4. Discussion

Molecular methods have been used to classify *Salmonella* isolates into serovars and subtypes for surveillance and epidemiological investigations. Although *S. enterica* serovar Schwarzengrund strains have been consistently isolated from food products [20], this is the first study to differentiate *S. enterica* serovar Schwarzengrund strains using different molecular techniques. We used different molecular methods (i.e., PFGE, MLST, CRISPR, and WGS), all of which have been commonly used for *Salmonella* source tracking [6,37], to differentiate *S. enterica* serovar Schwarzendrund intraserovar strains. The discriminatory power of these different typing methods was determined using 23 epidemiologically unrelated, and one related, *S. enterica* serovar Schwarzengrund strains from different veterinary sources, and measured by calculating Simpson’s index.

Fourteen PFGE types, two MLST types, eleven CRISPR types, and twenty WGS types were identified among 24 *S. enterica* serovar Schwarzengrund isolates used in this study. As SS07 and SS08 were from the same source, they were indistinguishable using all the tested methods. Moreover, SS07, SS08, SS10, SS15, SS16, SS17, and SS18 strains were clustered closely together into three different genetic groups among typing methods, suggesting the congruence between these methods. The highest Simpson’s index was obtained using WGS, followed by PFGE, CRISPR, and MLST. Our WGS, PFGE, and MLST results were consistent with those described previously [38,39,40]. In one study, PFGE differentiated 52 *S. enterica* serovar Enteritidis isolates into eight subtypes, while WGS differentiated the same isolates into 34 types, resulting in discriminatory values of 0.81 and 0.97, respectively [38]. Another study compared differentiation potential of PFGE and WGS across 55 *S. enterica* serovar Enteritidis isolates. PFGE differentiated 10 subtypes, whereas WGS further differentiated the isolates into 45 unique subtypes [40], highlighting the greater discriminatory power of WGS over PFGE. Conversely, ST96 and ST332 were recently reported as the dominant ST of *S. enterica* serovar Schwarzengrund isolates in Taiwan and were the only two MLST types observed in this study. Therefore, MLST typing showed no discriminatory power for *S. enterica* serovar Schwarzengrund. Similarly, a study of 30 *S. enterica* serovar Enteritidis isolates collected in Japan between 1973 and 2004 assigned all isolates to MLST 11, with no nucleotide differences in seven housekeeping genes. As genetic mutations accumulate slowly in housekeeping genes [41], they prevent MLST typing from thoroughly detecting or resolving relationships in closely related species.

A high degree of CRISPR polymorphism was observed in this study, suggesting that CRISPR loci might provide useful information for typing [42]. Like the majority of *Salmonella* serovars, *S. enterica* serovar Schwarzengrund has two CRISPR arrays, CRISPR1 and CRISPR2. These CRISPR arrays differ in spacer composition between closely related strains due to their strain-specific exposure histories to phages and plasmids [42]. CRISPR1 alleles were less polymorphic than CRISPR2, indicating stronger selective pressure for maintaining nucleotide integrity at this locus, which is consistent with previous studies [43,44]. In this study, the CRIPSR typing approach was feasible for subtyping *S. enterica* serovar Schwarzengrund isolates, although its discriminatory power is lower than that of PFGE. The isolate clustering based on PFGE was more similar to clustering by WGS than to clustering by CRISPR. Previous studies have suggested that CRISPR spacers are potentially involved in controlling plasmid- and phage-mediated horizontal gene transfer [45], and the frequent gain and loss of these spacers is due to homologous recombination [46]. Therefore, rapid diversification of spacers may lead to problems with CRISPR-based classification of *S. enterica* serovar Schwarzengrund strains.

WGS was found to be the most discriminatory method used in this study. PFGE has been considered the gold standard for bacterial typing, and has been successfully used to type *Salmonella* species from human, foods, and food-production animals, for over two decades [47]. Nevertheless, a transition from PFGE to WGS has occurred due to the rapid growth and decreasing cost of full genome sequencing over the past decade. Since WGS can examine complete or nearly complete bacterial genomes at a single-nucleotide level, it is not surprising that WGS provides the greatest level of discrimination between strains.

SNP and gene-by-gene approaches are the two most common methodologies for WGS typing of isolates (genomic MLST) [48]. Studies have shown that SNP and genomic MLST results are congruent and both approaches can analyze the outbreaks of foodborne illnesses [49]. However, a recent study that investigated two outbreaks using different WGS subtyping methods revealed that SNP-based analyses have the ability to confirm the occurrence of the outbreak while both cgMLST and wgMLST could neither differentiate outbreak-related *Salmonella* Typhimurium isolates from outbreak-unrelated isolates nor confirm the source of infection [50]. Consequently, WGS based on SNP was selected for further investigation.

Currently, genetic variant calling is based on the alignment of raw sequence reads against a reference genome to yield insights for SNP discovery [51]. However, bacteria are subject to constant evolutionary pressures that favor competition, resulting in events such as horizontal gene transfer and loss of genes and genome segments [52]. Moreover, a lack of complete and well assembled *S. enterica* serovar Schwarzengrund reference genome may cause biases due to mapping errors. Hence, the de novo assembly-based approach was used in this study.

After de novo assembly, the average nucleotide identity (ANI) and MUMi are the most used algorithms for taxonomic studies. While ANI calculates the proportion of DNA shared by two genomes [53], MUMi assesses the number of maximal unique and exact matches of a given minimal length shared by the two genomes [54]. However, previous studies using ANI have shown discrepancy with DNA–DNA hybridization analysis when intraspecies differences were assessed [55]. Moreover, when comparing subspecies, MUMi was more robust on intraspecies differentiation [32,56]. Therefore, MUMi was chosen over ANI for WGS analysis in this study. However, it is important to note that using different WGS analysis approaches or reference genomes results in different SNP profiles, which makes it difficult to compare results between studies. Moreover, a perceived limitation in this study is the de novo assembly from high coverage short (Illumina) reads. It has been shown that despite the use of sophisticated bioinformatic algorithms, short reads could miss some aspects in the identification of structural variants, the sequencing of repetitive regions, the phasing of alleles, and for distinguishing highly homologous genomic regions [57]. Long-read sequencing technologies, however, should offer improvements in the characterization of genetic variation and regions [57]. In the future, there is a need for an international standardization of WGS analysis to allow for accurate comparisons of results across laboratories.

## 5. Conclusions

This study compares the utility of four subtyping methods to differentiate *S. enterica* serovar Schwarzengrund isolates from different veterinary sources. Among genotyping approaches, WGS was shown to be the most discriminatory method to subtype *S. enterica* serovar Schwarzengrund. However, this study only uses a small number of *S. enterica* serovar Schwarzengrund isolates from sporadic cases, and, therefore, is not comprehensive enough for surveillance and outbreak investigations. Moreover, de novo assembly from short reads could miss some aspects in the identification of structural variants, the sequencing of repetitive regions, the phasing of alleles, and for distinguishing highly homologous genomic regions. Further validation is required by using long-read sequencing technologies and a larger number of *S. enterica* serovar Schwarzengrund isolates that have been epidemiologically linked to the same source in order to establish the epidemiological concordance of the data.

## Figures and Tables

**Table 1:** *S. enterica* serovar Schwarzengrund strains used in this study.

Sample ID	Source	Isolation Year
SS01	Duck	2000
SS02	Pig	2000
SS03	Dog	2003
SS04	Pig	2003
SS05	Broiler	2005
SS06	Pig	2006
SS07	Broiler (Farm A; internal control)	2008
SS08	Broiler (Farm A)	2008
SS09	Broiler (Farm K)	2008
SS10	Pet food	2008
SS11	Broiler	2009
SS12	Broiler	2010
SS13	Crested Goshawk	2011
SS14	Moorhen	2011
SS15	Turkey	2012
SS16	Duck	2012
SS17	Pig (Farm B)	2012
SS18	Pig (Farm C)	2012
SS19	Turkey (Farm D)	2012
SS20	Turkey (Farm E)	2012
SS21	Pig	2013
SS22	Broiler	2014
SS23	Pig	2015
SS24	Broiler	2018

**Table 2:** Comparison of different subtyping methods for *S. enterica* serovar Schwarzengrund isolates.

Method	No. of Types	Discriminatory Power	95% Confidence Interval
PFGE	14	0.938	(0.937, 0.939)
MLST	2	0.463	(0.455, 0.471)
CRISPR	11	0.906	(0.905, 0.907)
WGS	20	0.982	(0.982, 0.982)

## Data Availability

The WGS data used in this study were deposited to the NCBI database under BioProject accession number PRJNA635494.

## Supplementary Materials

The following are available online at https://www.mdpi.com/article/10.3390/microorganisms9102046/s1. Table S1. Primers used in this study. Table S2. List and nucleotide sequence of CRISPR spacers.
