# Supplementary material for: Comparison of Conventional Molecular and Whole-Genome Sequencing Methods for Differentiating Salmonella enterica Serovar Schwarzengrund Isolates Obtained from Food and Animal Sources
Source: Microorganisms. 2021 Sep 28;9(10):2046. doi: 10.3390/microorganisms9102046 (PMC8540620; doi:10.3390/microorganisms9102046)
Supplement: Supplementary file 1 [file microorganisms-09-02046-s001.zip › microorganisms-1401768-supplementary.pdf]

Table S1. Primers used in this study

Name	Forward (5'→3')	Reverse (5'→3')
*aro*C	GGCACCAGTATTGGCCTGCT	CATATGCGCCACAATGTGTTG
*dna*N	CCGATTCTCGGTAACCTGCT	CCATCCACCAGCTTCGAGGT
*hem*D	GTGGCCTGGAGTTTTCCACT	GACCAATAGCCGACAGCGTAG
*his*D	GTCGGTCTGTATATTCCCGG	GGTAATCGCATCCACCAAATC
*pur*E	CGCATTATTCCGGCGCGTGT	CGCGGATCGGGATTTTCCAG
*suc*A	AGCACCGAAGAGAAACGCTG	GGTTGTTGATAACGATACGTAC
*thr*A	ATCCCGGCCGATCACATGAT	CTCCAGCAGCCCCTCTTTCAG
CRISPR1[a]	GATGTAGTGCGGATAATGCT	GGTTTCTTTTCTTCCTGTTG<br>GATGATATGGCAACAGGTTT
CRISPR2	ACCAGCCATTACTGGTACAC	ATTGTTGCGATTATGTTGGT

[a]The two reverse primers of CRISPR1 were added together with the forward primer to amplify CRISPR1

Table S2. List and nucleotide sequence of CRISPR spacers

Spacer No.	CRISPR1	CRISPR2	SS13
1	TTTTTAGAGGTGTACACACTGTCGGCCTTTTG	TCGTAGTCTTCCGGGTAGGCGTTTTCATCCGG	CGTTGCTGGCTATTCGAACTAACCTGATTGTT
2	CACCTTAGCGACGAAATTAAAACCGAGCTCAC	TCTTTATCAGCTAACCATTTCCAGAACTCGTC	GCCTCAGCATTGTCATAACCCCACGCCTGGCA
3	GCTTTGCCCCATGCGTCCAGATCTTCAACGGG	GCGAGCGTTTTGGGTGGCACAAAATCCCCGAG	CGGCTCGACCGGGAGCTTGCCGGCATCGTGGA
4	AGAAAATTATCACTGACGAGTGACAATAACTC	GTAACTTAATAAGGTCCTGGACATTAATTTTG	GCAACTTCTTATATGTAAAAAACCGTTATGAT
5	GGAGGTGATCCGGGATTTAAACAAAATGATTA	AGTGCCGATGCGCGGTATCTGGGGAATTTCGC	TCCGGGGAAAGAACTCGCCTCGTCCAGCGTCA
6	GGTTTACAGACCTCAATAAAAAGACGGCAGGC	ATAAAGGTCGGGGACGATATTGTTACCTGGTG	CGGCGGCGGACGCCGCTCAGATAGCCAAAACC
7	GTCAGCGCCGGCGATTGGGCCGCCGATTTTGT	GGTGGAATCGCGCAAAACTTTGTTTGTTTCCG	TATACAGGATTCTCACACTTTGGTTTGGTCGA
8	AATGCAATTTCCTGACTCCCCTAACCTCAAGG	GGGTACTGACAATAGTACAAATAAAAAAAACG	CGTTTTTTTTATTTGTACTATTGTCAGTACCC
9	AAGTACCGGCAAGTGTGGTAGCGAAGGTTATG	TCGACCAAACCAAAGTGTGAGAATCCTGTATA	CGGAAACAAACAAAGTTTTGCGCGATTCCACC
10	GCGCTTGATCTCCCGTTATCTAAGGGCCAATC	GGTTTTGGCTATCTGAGCGGCGTCCGCCGCCG	CACCAGGTAACAGTATCGTCCCCGACCTTTAT
11	GCATCGGTGGCGCTGGCCAGTTCAAAAATACG	TGACGCTGGACGAGGCGAGTTCTTTCCCCGGA	GCGAAATTCCCCAGATACCGCGCATCGGCACT
12	CTCAAACGAACGCAGTATTTGATCGGATGTTT	ATCATAACGGTTTTTTACATATAAGAAGTTGC	CAAAATTAATGTCCAGGACCTTATTAAGTTAC
13		TCCACGATGCCGGCAAGCTCCCGGTCGAGCCG	CTCGGGGATTTTGTGCCACCCAAAACGCTCGC
14		TGCCAGGCGTGGGGTTATGACAATGCTGAGGC	GACGAGTTCTGGAAATGGTTAGCTGATAAAGA
15		AACAATCAGGTTAGTTCGAATAGCCAGCAACG	CCGGATGAAAACGCCTACCCGGAAGACTACGA
16		GGGTTCGTTAGCGGGATCTGGTTGCTGCGCCA	
